# New Strategies for Conservation of Gentile di Puglia Sheep Breed, an Autochthonous Capital of Millennial Tradition in Southern Italy

**DOI:** 10.3390/ani13142371

**Published:** 2023-07-20

**Authors:** Letizia Temerario, Davide Monaco, Antonella Mastrorocco, Nicola Antonio Martino, Sándor Cseh, Giovanni Michele Lacalandra, Elena Ciani, Maria Elena Dell’Aquila

**Affiliations:** 1 Department of Biosciences, Biotechnologies & Environment, University of Bari Aldo Moro, Strada per Casamassima km 3, Valenzano, 70010 Bari, Italy; antonella.mastrorocco@uniba.it (A.M.); nicola.martino@uniba.it (N.A.M.); elena.ciani@uniba.it (E.C.); mariaelena.dellaquila@uniba.it (M.E.D.); 2 Department of Veterinary Medicine, University of Bari Aldo Moro, Strada per Casamassima km 3, Valenzano, 70010 Bari, Italy; davide.monaco@uniba.it (D.M.); giovannimichele.lacalandra@uniba.it (G.M.L.); 3 Department of Obstetrics and Food Animal Medicine Clinic, University of Veterinary Medicine, István St. 2, 1078 Budapest, Hungary; cseh.sandor@univet.hu

**Keywords:** Gentile di Puglia sheep breed, endangered, conservation, ultrasound, pregnancy, oocyte, vitrification, in vitro maturation, oocyte bioenergetic-oxidative status

## Abstract

**Simple Summary:**

In recent years, due to industry and market preferences, local animal breeds have been exposed to genetic erosion and extinction risk. Effective strategies for their recovery and conservation are needed. Gentile di Puglia (GdP) is an autochthonous sheep breed, typical of Southern Italy, with an aptitude for wool, meat, and milk production and considerable historical and cultural value. The development of a GdP gamete and embryo cryobank could help to support the numerical reimplementation of this population. Animal germplasm conservation is mainly performed through sperm cryopreservation, whereas strategies on the female side are developed in a limited way. In this study, the dual purpose of monitoring the reproductive efficiency in one pilot GdP farm in the Apulia region and setting up a cryopreservation protocol, by vitrification, of immature cumulus-oocyte complexes (COCs) recovered from pre-pubertal lambs, followed by an analysis of their in vitro maturation potential and bioenergetic-oxidative status, was pursued. The results indicated that traditional reproductive management leads to progressive offspring reduction and that ex situ biotechnological conservation strategies, through immature oocyte vitrification and in vitro maturation, can support in situ conservation, leading to in vitro embryo production and transfer.

**Abstract:**

Gentile di Puglia (GdP) is an autochthonous sheep breed of Southern Italy included among ovine breeds threatened by genetic erosion and extinction risk, which have been given attention by local and international institutions, thus emphasizing the need for germplasm conservation actions. In the present study, two assisted reproduction approaches, finalized for GdP conservation, were performed: (1) on-farm reproductive efficiency evaluation, expressed as pregnancy rate (PR), twin pregnancy rate (tPR), and body condition score (BCS), for three consecutive breeding cycles and (2) pre-pubertal lambs’ immature cumulus–oocyte complex (COC) retrieval, vitrification, in vitro maturation (IVM), and assessment of meiotic stage and bioenergetic-oxidative status compared with those of other Italian and European commercial breeds. PR and tPR were progressively reduced over time. In all clinical examination times, BCS was significantly lower in nonpregnant ewes compared with pregnant ones. Fresh GdP pre-pubertal lamb COCs achieved meiotic maturation and showed healthy bioenergetic–oxidative status after IVM. Vitrification reduced the oocyte maturation rate in all groups. However, mature oocytes retained their cytoplasmic maturity, expressed as a mitochondria distribution pattern and activity, indicating promising developmental competence. In conclusion, clinical- and biotechnological-assisted reproduction approaches can support conservation strategies of GdP and other local sheep breeds in Southern Italy.

## 1. Introduction

At a global level, many local breeds are at risk of genetic erosion and extinction due to their very localized distribution, replacement with more productive commercial breeds, and high inbreeding rates [1]. In Southern Italy, the rearing of local breeds is strongly linked to the territory for gastronomic, historical, and cultural aspects. This provides inimitable typical foods and leads to emerging and broad industry and market outcomes [2]. However, current agricultural policies tend to not effectively support rearing programs of local breeds, and farmers are turning their interest to more productive breeds, including those from other countries. This has led to a significant reduction in animal number with consequent genetic erosion and, in some cases, to the risk of extinction with a negative impact not only on typical local products that characterize and qualify the regional agro-farming activities but also and mainly on the ecosystemic services that the breeding activities provide to the communities, particularly in marginal areas.

Gentile di Puglia (GdP), literally “Gentle Apulian” because of its fine wool, is an autochthonous sheep breed of millenary origins on the Southern Italy territory, mainly bred in the area of Foggia (Tavoliere delle Puglie and Monti Dauni) [2,3]. GdP is characterized by its aptitude for wool, meat, and milk production and has been shown to be genetically adapted and resilient to the environmental conditions of marginal areas where it is normally reared [4]. In the last 50 years, due to the wool market crisis and indiscriminate crossbreeding, a significant numerical contraction has been observed in this local ovine breed [3]. Indeed, the last census of GdP population for the year 2022, carried out by ASSONAPA (Associazione Nazionale della Pastorizia; Italian Pastoral Farming Association), reported that the number of heads of this breed is very limited in Italy, consisting of approximately 4000 animals, including about 250 rams and 3500 ewes (P. Fresi, personal communication). Therefore, GdP needs to be preserved for the productive, historical, and cultural value that it represents for the Southern Italy community and worldwide biodiversity.

The conservation of genetic animal resources can be performed in situ and ex situ, depending on whether animals are kept within their natural environments or production systems [5]. As an in situ approach, pregnancy diagnosis (PD) is a key aspect of flock management. PD during early gestation allows sheep breeders to make important economic decisions. These include identifying nonpregnant or sick ewes for treatment, rebreeding or culling, and improving the nutritional plane of pregnant ones to optimize offspring weights, prevent pregnancy toxemia, and increase milk production [6]. Transabdominal B-mode ultrasound is a quick, non-invasive, and accurate reproductive technology used in small ruminants for PD, determination of fetal number and viability, and detection of pathological condition [7]. Ex situ strategies can be divided into in vivo and in vitro, depending on whether the animal germplasm is kept in the form of live animals or cryopreserved through a gene-banking strategy [5]. By setting up a cryobank, it is possible to collect and cryopreserve different kinds of cells and tissues, such as semen, oocytes, embryos, ovarian/testicular tissue, somatic, stem, and induced pluripotent stem cells, and, thanks to the advancements in reproductive biotechnologies, it is possible to obtain live animals from these cells in different times and places [1,8,9]. Gene banking of animal genetic resources is a strategic priority of the Global Plan of Action for Animal Genetic Resources, which was developed and adopted by the Food and Agriculture Organization (FAO) Member Nations (2007), and of the Sustainable Development Goals (SDGs), which were adopted by the United Nations (UN) (2015) with the goal of achievement by 2030, specifically under Target 2.5 of maintaining the biodiversity of plants and animals. In Italy, there is not yet a national animal genetic resources gene bank “https://www.eugena-erfp.net/en/ (accessed on 7 June 2023)”, and the set-up of these initiatives is still at an initial stage and localized at the regional level. In 2023, the Animal Germplasm Cryobank created by the Institute of Agricultural Biology and Biotechnology of the National Research Council (IBBA-CNR), in collaboration with the Department of Veterinary Medicine and Animal Sciences of the University of Milan (DIVAS-UNIMI), has been registered to the Italian National Registry of biodiversity for agriculture and food, as an ex situ conservation center and/or Germplasm Banks for genetic resources, through Ministerial Decree No. 207219, dated 17 April 2023. Regarding Apulia, regional law n.39 was approved in 2013 with the aim of conserving zootechnical autochthonous genetic resources that are threatened by genetic erosion or risk of extinction and for which environmental, cultural, scientific, and economic interests exist. In this context, the proposal for a cryobank of GdP genetic resources is innovative, as it meets the need to seek effective strategies for the recovery, conservation, and use of genetic resources of native animal breeds aiming to obtain sustainable products of intra- and extra-regional interest with technologies that guarantee quality, traceability, and safety.

Oocyte cryopreservation offers the possibility of storing and spreading female germplasm from endangered breeds and individuals of great value. This is a versatile tool, in combination with selected semen samples, allowing offspring to be created that will fill a current need at the time of use [9,10]. Female gametes can be retrieved from live animals or isolated ovaries after slaughter or unexpected death. Slow freezing and vitrification are the two main techniques developed for oocyte cryopreservation. However, the success rate is still challenging because of the large oocyte size, low surface-to-volume ratio, cytoskeleton structure, lipid content, and meiosis stage [9,10].

The cryopreservation of immature oocytes at the germinal vesicle (GV) stage allows the obtainment of a significant number of female gametes without hormonal stimulation or in vitro maturation (IVM) right after collection. Moreover, through the vitrification procedure, it is possible to preserve them directly in farms located in marginal areas, where equipped laboratories are too far or not available. Immature oocytes are usually cryopreserved as cumulus–oocyte complexes (COCs) because cumulus cells (CCs) are crucial for oocyte meiotic resumption and developmental competence [11]. However, IVM is challenging for cryopreserved immature COCs due to membrane damage to CCs [12] and their physical/functional detachment from the oocyte, caused by transzonal projections’ sensitivity to cryoprotectants and low temperatures [12,13]. In the last 20 years, the vitrification/warming of immature COCs from adult subjects has been applied to several domestic large (sheep [12,14,15,16,17,18,19,20,21,22], cow [23,24,25,26,27], buffalo [28,29,30], goat [31], pig [32,33,34,35], horse [36,37,38,39,40,41], donkey [42]) and small (cat [43,44,45,46,47,48], dog [49]) animal species, with various results, depending on intrinsic species-specific oocyte features. Although the results of IVM, embryo development, and blastocyst rate following immature COC vitrification are still less satisfactory compared to fresh COCs, live births have been reported in bovine [50], swine [33], equine [38,41], and domestic feline [43] species.

Immature COC vitrification from pre-pubertal animals offers additional interesting features. In Southern Italy, the high consumption of lamb meat, for traditional and cultural reasons, allows germplasm to be obtained and recovered, which would otherwise be discarded, from a significant quantity of slaughtered pre-pubertal ovaries. Moreover, the use of this kind of ovaries allows the generation interval to be reduced, the rate of genetic gain to be increased, and more COCs to be obtained than from adult ewe ovaries [51,52]. To the best of our knowledge, there is only one study focusing on immature COC vitrification using pre-pubertal lamb ovaries [53] and only one aimed at local breed conservation in the autochthonous Vietnamese Ban Pig breed [54].

According to the previous considerations, since the use of high-quality germplasm is closely linked with the tracking of productive and reproductive data, the first aim of the present study was to monitor the reproductive efficiency of a GdP farm with a traditional breeding system. Then, with the aim of developing a cryobank of GdP germplasm, in vitro reproductive potential and cryotolerance after the vitrification of pre-pubertal immature COCs from a GdP local sheep breed, assessed in terms of the maturation rate and bioenergetic-oxidative status, was investigated. Data were compared with those obtained from oocytes recovered from Italian and European commercial-breed lamb ovaries.

## 2. Materials and Methods

### 2.1. Routine Veterinary Checkups

#### On-Farm Evaluation of Reproductive Efficiency

**Figure 1:**
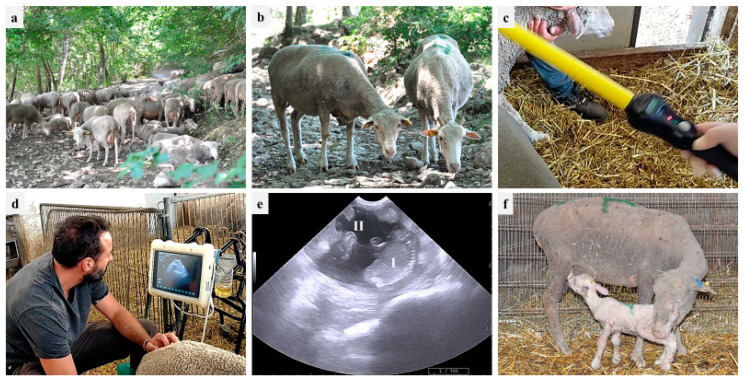
On-farm reproductive monitoring in one Gentile di Puglia (GdP) flock: (**a**) flock in the pasture; (**b**) two GdP ewes; (**c**) ruminal bolus reading; (**d**) transabdominal ultrasound; (**e**) pregnancy diagnosis based on fetus (I) and cotyledon (II) identification; (**f**) suckling GdP lamb.

During 2021 and 2022, PDs were performed in one farm (partner of the PhD Program 2020 FSC—Piano Stralcio “R&I 2015–2017”, University of Bari Aldo Moro, Italy), located in the area of Monti Dauni (Southern Italy), a place with a historical vocation for GdP-breeding activities. The farm was established in the 20th century, and about six hundred GdP sheep, including around 20 rams, are kept through family-based traditional breeding systems (pasture plus barn-concentrate integration, if needed). The owner reported that, besides the male effect, no assisted reproductive technologies (synchronization, artificial insemination, PD) are implemented in the farm. According to traditional local consumption, 60-day-old lambs are sold three times per year: (i) Easter time, (ii) mid-August, and (iii) Christmas time. Therefore, breeding cycles are scheduled in September, January, and May, respectively. PDs were performed using an 8–10 MHz convex probe (MyLab, Esaote ultrasound device), starting from 40 days after the removal of rams from the ewe flock. The transabdominal approach was used: ewes were restrained in stanchions similar to cattle chutes with headgates, and the probe was positioned at the right inguinal level, near the base of the udder. The observations of a fluid-filled uterus with placentomes and at least one fetus were considered positive signs of pregnancy [7,55]. Figure 1 shows GdP ewes during the different stages of the PD procedure and lambs. In addition, the ewes’ body condition score (BCS) was evaluated (through the manual palpation at the lumbar vertebrae) in pregnant and nonpregnant ewes, providing a score from 0 to 5 and considering 2.75–3 as the ideal value [56].

### 2.2. In Vitro Study

#### 2.2.1. Chemicals

All chemicals for in vitro cultures and analyses were purchased from Sigma-Aldrich (Milan, Italy), unless otherwise indicated.

#### 2.2.2. Collection of Ovaries and COC Retrieval

Ovaries from pre-pubertal lambs (less than 6 months of age) were recovered at local slaughterhouses from animals subjected to routine veterinary inspection in accordance with the specific health requirements stated in Council Directive 89/556/ECC and subsequent modifications. Ovaries were transported to the laboratory at room temperature within 4 h of collection. For COC retrieval, ovaries underwent the slicing procedure [57]. The follicular contents were released in sterile Petri dishes containing phosphate-buffered saline (PBS) and observed under a Nikon SMZ18 stereomicroscope equipped with a transparent heating stage set up at 38.5 °C (Okolab S.r.l., Napoli, Italy). Only COCs with at least three intact cumulus cell layers and homogenous cytoplasm were selected for culturing [58].

#### 2.2.3. Vitrification and Warming Procedures

**Figure 2:**
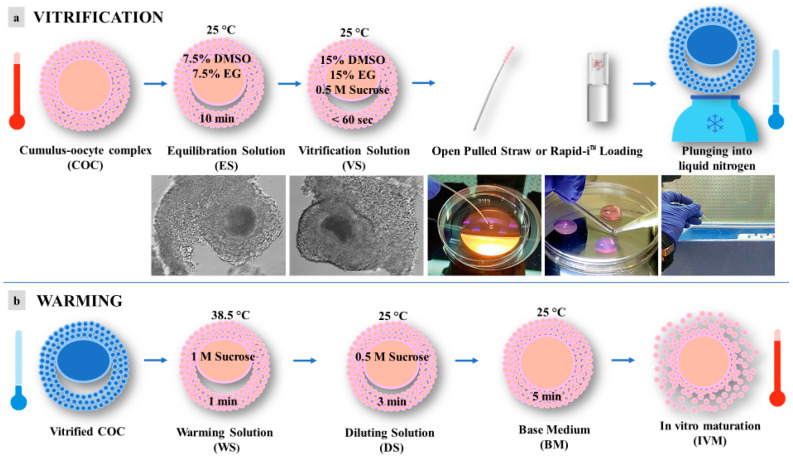
Schematic representation of vitrification protocol for pre-pubertal lambs’ immature cumulus-oocyte complexes (COCs): (**a**) vitrification; (**b**) warming. DMSO = dimethyl sulfoxide; EG = ethylene glycol.

Vitrification and warming were performed according to the procedures reported by dos Santos-Neto et al., 2020 [21], with some modifications (Figure 2). In order to counteract cryoprotectant toxicity, all vitrification media were used at room temperature, except for the warming solution, which was used at 38.5 °C. Briefly, selected immature COCs, in groups of 5, were incubated for 10 min in 300 μL drops of equilibration solution (ES) containing 7.5% (*v*/*v*) ethylene glycol (EG) and 7.5% (*v*/*v*) dimethyl sulfoxide (DMSO) and dissolved in base medium (BM), containing 20% (*v*/*v*) fetal calf serum (FCS) added to Hepes-buffered TCM 199. After equilibration, the oocytes were placed into 300 μL drops of vitrification solution (VS) containing 15% (*v*/*v*) EG, 15% (*v*/*v*) DMSO, and 0.5 mol/L sucrose dissolved in BM. Oocyte vitrification was performed in two steps/drops in less than 60 s. After that, oocytes were loaded into an Open Pulled Straw (OPS) (Minitube) or onto a Rapid-i™ Kit (Vitrolife) with a minimum volume (e.g., <0.1 μL) and plunged quickly into liquid nitrogen. Warming was performed by submerging the vitrification device directly into warming solution (WS) containing 1 mol/L sucrose dissolved in BM at 38.5 °C for 1 min. Warmed oocytes were transferred to a 300 μL drop of dilution solution (DS) with BM plus sucrose 0.5 mol/L for 3 min and then washed twice in 300 μL drops of BM for 5 min.

#### 2.2.4. In Vitro Maturation (IVM)

IVM medium was prepared based on TCM-199 medium with Earle’s salts. It was buffered with 5.87 mmol/L HEPES and 33.09 mmol/L sodium bicarbonate and supplemented with 0.1 g/L L-glutamine, 2.27 mmol/L sodium pyruvate, calcium lactate pentahydrate (1.62 mmol/L Ca^2+^, 3.9 mmol/L Lactate), 50 μg/mL gentamicin, 20% (*v*/*v*) fetal calf serum (FCS), 10 μg/mL of porcine follicle stimulating hormone and luteinizing hormone (FSH/LH; Pluset^®^, Calier, Balcellona, Spain) [59], and 1 μg/mL 17β estradiol [57]. IVM medium was pre-equilibrated for 1 h under 5% CO_2_ in air at 38.5 °C, then loaded (400 μL/well) in a 4-well dish (Nunc Intermed, Roskilde, Denmark) and covered with pre-equilibrated lightweight paraffin oil. In each experiment, 20–25 COCs/well were added to a 4-well dish and cultured for 22–24 h at 38.5 °C under 5% CO_2_ in air.

#### 2.2.5. Assessment of Cumulus Expansion and Oocyte Denuding

After IVM, COCs were recovered, and cumulus expansion was checked. COCs showing cumuli with continuous edges, consisting of cells in close contact each other, were classified as compact, whereas cumuli showing discontinuous edges following cell detachment and the production of a viscous extracellular matrix were classified as expanded. Even though the cumulus expansion does not fully ensure that maturation is achieved, the percentage of expanded COCs was recorded because it represents the response of immature COCs to the presence of gonadotropins in the culture medium. For oocyte denuding, COCs underwent cumulus cell removal by incubation in TCM-199 with 20% FCS containing 80 IU hyaluronidase/mL and aspiration in and out of finely drawn glass pipettes. Denuded oocytes were evaluated for their meiotic stage, and mature ones were used to assess bioenergetic/oxidative status.

#### 2.2.6. Oocyte Mitochondria and ROS Staining

Oocytes were washed three times in PBS with 3% BSA and incubated for 30 min in the same medium containing 280 nmol/L MitoTracker Orange CMTM Ros (Thermo Fisher Scientific, Waltham, MA, USA) at 38.5 °C under 5% CO_2_ in air. After incubation with MitoTracker, oocytes were washed in PBS with 0.3% BSA and incubated for 15 min, at 38.5 °C under 5% CO_2_, in air in the same medium containing 10 µmol/L 2, 7—dichlorodihydrofluorescein diacetate (H_2_DCF-DA) to detect the dichlorofluorescein (DCF) and localize intracellular sources of ROS [60]. After incubation, oocytes were washed in PBS without BSA and fixed overnight at 4 °C in 4% paraformaldehyde (PFA) solution in PBS [61]. Particular attention was applied to avoid sample exposure to the light during staining and fixing procedures and to reduce photobleaching.

#### 2.2.7. Oocyte Nuclear Chromatin Evaluation

To evaluate oocytes’ nuclear chromatin, after the fixation in 4% PFA in PBS, oocytes were stained with 2.5 µg/mL Hoechst 33258 in 3:1 (*v*/*v*) glycerol/PBS mounted on microscope slides with coverslips, sealed with nail polish, and kept at 4 °C in the dark until observation. Slides were examined under the epifluorescence microscope (Nikon Eclipse 600; Nikon Instruments, Firenze; ×400 magnification) equipped with a B-2A (346 nm excitation/460 nm emission) filter. Oocytes were evaluated in relation to their meiotic stage and classified as germinal vesicle (GV), metaphase to telophase I (MI to TI), and MII with the first polar body (PB) extruded [62]. Oocytes showing either multipolar meiotic spindle, irregular chromatin clumps, or the absence of chromatin were considered abnormal [63].

#### 2.2.8. Assessment of Mitochondrial Distribution Pattern and Intracellular ROS Localization

Oocytes at the MII stage were observed at ×600 magnification in oil immersion with a Nikon C1/TE2000-U laser scanning confocal microscope (Nikon Instruments, Firenze, Italy). A 543 nm helium/neon laser and a G-2A filter were used to detect the MitoTracker Orange CMTM Ros (551 nm excitation and 576 nm emission). A 488 nm argon ion laser and a B-2A filter were used to detect DCF (495 nm excitation and 519 nm emission). Scanning was conducted with 25 optical sections from the top to the bottom of the oocytes, with a step size of 0.45 µm to allow for 3D distribution analysis. The mitochondrial distribution pattern was evaluated on the basis of previous studies: (1) finely granular, with small mitochondria aggregates spread throughout the cytoplasm, typical of immature oocytes; (2) perinuclear and subcortical (P/S) distribution of mitochondria forming large granules, which is an indicator of cytoplasmic maturity; and (3) abnormal, with irregular distribution of mitochondria [61]. Concerning intracellular ROS localization, oocytes with intracellular ROS distributed throughout the cytoplasm, together with areas/sites of mitochondria/ROS overlapping, were considered healthy.

#### 2.2.9. Quantification of Bioenergetic/Oxidative Parameters

In each individual oocyte, MitoTracker and DCF fluorescence intensities were measured at the equatorial plane and at the excitation/emission, as described above, using the EZ-C1 Gold Version 3.70 image analysis software platform for Nikon C1 confocal microscope. A circular area was drawn in order to measure only the region including cell cytoplasm. The fluorescence intensity within the programmed scan area was recorded and plotted against the conventional pixel unit scale (0–255). Mitochondrial activity and intracellular ROS levels were recorded as MitoTracker Orange CMTM Ros and DFC fluorescence intensity in arbitrary densitometric units (ADUs). Parameters related to fluorescence intensity, such as laser energy, signal detection (gain), and pinhole size, were maintained at constant values for all measurements. The degree of mitochondria/ROS colocalization, reported as a biomarker of healthy oocytes [61,62], was quantified by the overlap coefficient between MitoTraker Orange CMTM Ros and the DCF fluorescence intensity signals.

#### 2.2.10. Statistical Analysis

The percentage of pregnant ewes and those carrying out twin pregnancies, in the three examined periods (July 2021, November 2021, and February 2022), were compared through the Chi-Square test. Unpaired Student’s *t*-test was performed to compare, at each time period, the BCS between pregnant and nonpregnant ewes. One-way ANOVA (followed by Tukey’s Multiple Comparison Test) was performed to compare the BCS in pregnant and nonpregnant ewes in the three time periods.

The proportions of oocytes showing different chromatin configurations and mitochondria distribution patterns were compared between groups using the Chi-square test. Mitochondria and ROS quantification analysis was conducted on oocytes at the MII stage. Data (mean ± standard deviation (s.d.) of bioenergetic parameters) were compared using the unpaired Student’s *t*-test or one-way analysis of variance ANOVA followed by Tukey’s Multiple Comparison Test, according to comparison groups. Differences with *p* < 0.05 were statistically significant.

## 3. Results

### 3.1. On-Farm Evaluation of Reproductive Efficiency

The pregnancy rate (PR), twin pregnancy rate (tPR), and BCS of ewes reared at the farm at different evaluation times are reported in Table 1. Between months, significant reductions were found in PR and tPR. The BCS was significantly lower in nonpregnant animals compared with pregnant ones, in all time groups. Moreover, it was significantly higher in the last examined period (February 2022), as compared to the first one (July 2021), in both the pregnant and the nonpregnant groups.

### 3.2. GdP Pre-Pubertal Lamb COCs Achieve Meiotic Maturation after IVM

**Figure 3:**
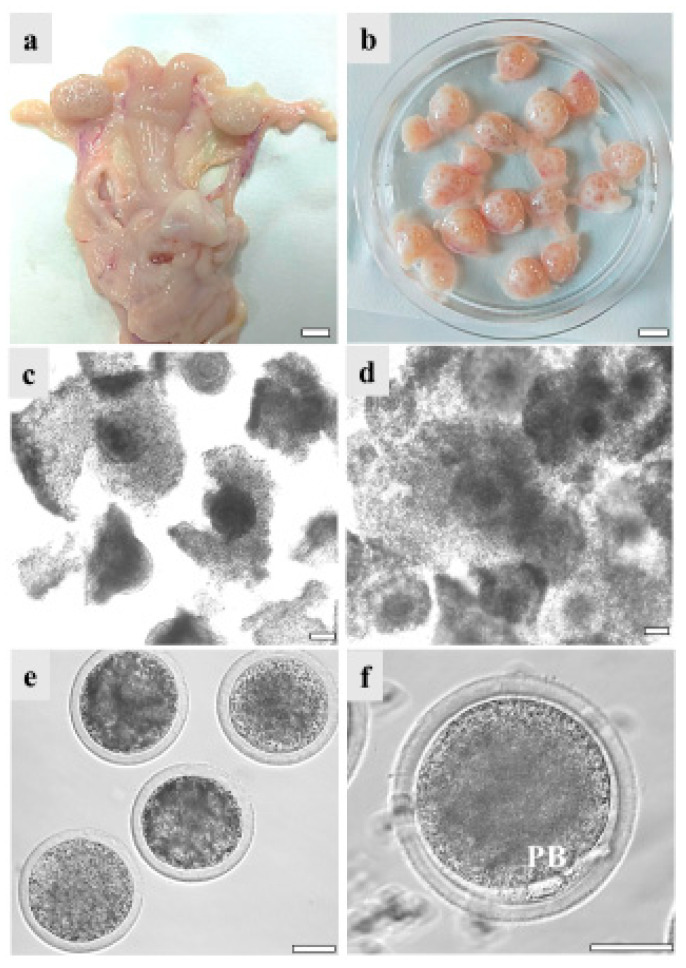
GdP female reproductive system specimens: (**a**) female reproductive tract with the two uterine horns, oviducts, and ovaries. Scale bar represents 1 cm; (**b**) ovaries isolated for cumulus–oocyte complex (COC) retrieval. Scale bar represents 1 cm; (**c**) COCs with compact or (**d**) expanded cumuli observed under inverted-phase contrast microscopy before and after 22–24 h IVM. Scale bars represent 40 μm; (**e**) denuded oocytes. Scale bar represents 40 μm. (**f**) Matured oocyte with the first polar body (PB) extruded. Scale bar represents 40 μm.

COCs recovered from the ovaries of GdP pre-pubertal lambs, reared and slaughtered in Apulia region, underwent IVM in order to evaluate their in vitro developmental potential. Data were compared with those of Italian (Comisana, Sardinian, and mixed breeds) and European (Merino and mixed breeds from Hungary and France) commercial cosmopolite sheep populations. Regardless of the sheep breed, pre-pubertal lamb ovary size ranged between about 0.5 and 1.5 cm showing follicles in different stages of growth, from 1 mm to approximately 5 mm. On the other hand, differences were found in the number of follicles and the COC recovery rate. Indeed, from GdP and Italian commercial breeds’ ovaries, it was possible to isolate around 20 good-quality COCs/ovary compared to 10 from the European ones. Pre-pubertal lamb COCs were analyzed in seven to eight independent IVM runs, followed by staining with Hoechst 33258 for nuclear chromatin evaluation. GdP COCs achieved cumulus expansion at significantly higher rates compared with other breeds (*p* < 0.05 and *p* < 0.001 with European and Italian populations, respectively; Table 2). Moreover, they achieved significantly higher maturation rates, showing the second metaphase plate and the first PB extruded, in comparison with the other sheep populations (*p* < 0.05 and *p* < 0.001 for European and Italian, respectively). Correspondingly, the percentage of oocytes that remained arrested at the GV stage was significantly reduced in GdP compared with other breeds (*p* < 0.001 and *p* < 0.00001, for European and Italian, respectively). Figure 3 shows specimens of the reproductive system of a GdP pre-pubertal lamb: a female reproductive tract with the two uterine horns, oviducts, and ovaries; ovaries showing several developing follicles isolated for COC retrieval; COCs with compact or expanded cumulus observed before and after 22–24 h IVM under inverted phase contrast microscopy; and denuded oocytes and a matured oocyte with the first PB extruded in the perivitelline space.

### 3.3. GdP Pre-Pubertal Lamb MII Oocytes Show Healthy Bioenergetic/Oxidative Status after IVM

**Figure 4:**
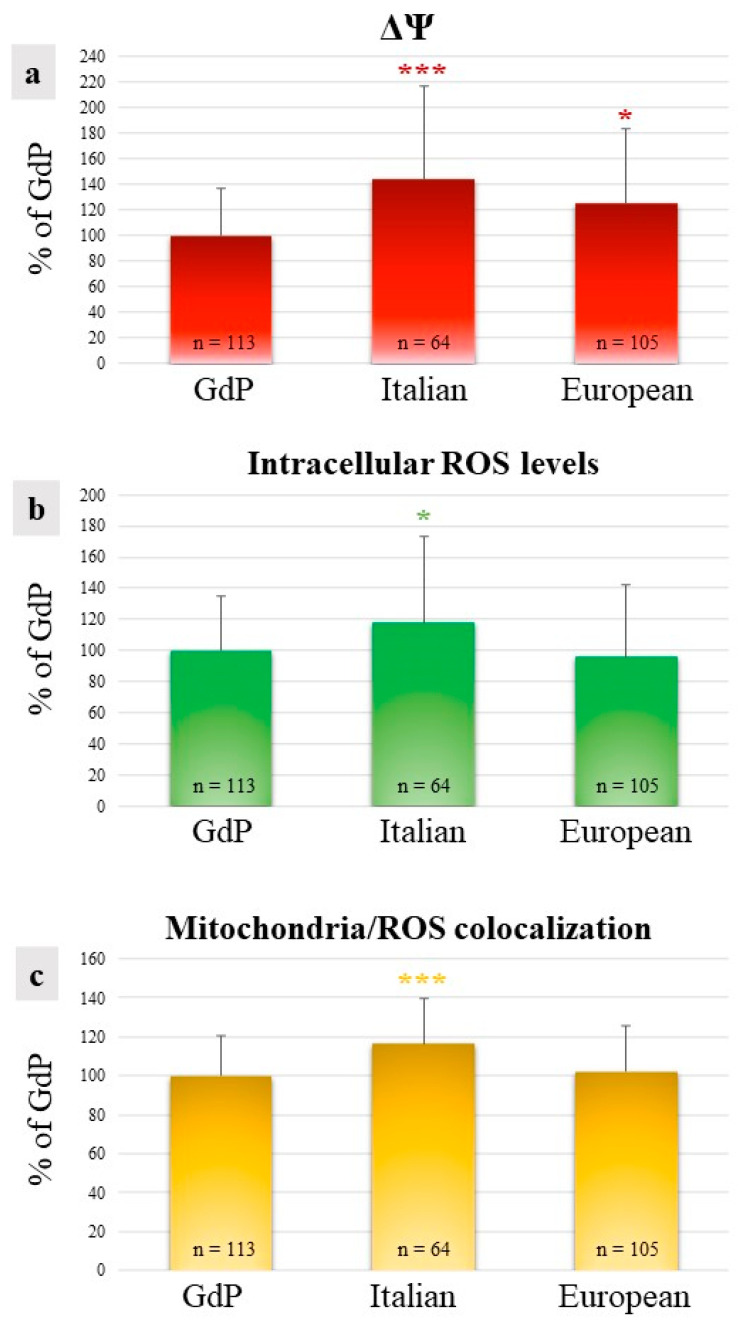
Quantification data of (**a**) mitochondrial membrane potential (ΔΨ), (**b**) intracellular reactive oxygen species (ROS) levels, and (**c**) mitochondria/ROS colocalization in GdP MII oocytes compared with Italian and European ones. Values are presented as percentages of the signal of GdP samples. Means ± SD of fluorescence intensity of the MitoTracker Orange CMTM Ros, DCF, and overlap coefficient are presented. The numbers of analyzed oocytes per sheep population are indicated at the bottom of each bar. One-way analysis of variance (ANOVA) followed by Tukey’s multiple comparison test: * *p* < 0.05; *** *p* < 0.001.

In GdP MII oocytes obtained after IVM, qualitative and quantitative parameters of the bioenergetic/oxidative status were analyzed as a measure of oocytes’ cytoplasmic maturity and competence to undergo fertilization and development. The percentages of MII oocytes showing a heterogeneous perinuclear and subcortical mitochondrial distribution pattern (P/S) did not vary among sheep population, indicating that a good rate of GdP oocytes, like those of other sheep populations, reached cytoplasmic maturity in the culture system used (Table 2). The mitochondrial membrane potential (ΔΨ) in GdP MII oocytes was significantly lower in comparison to other sheep populations (*p* < 0.05 and *p* < 0.001 for European and Italian, respectively; Figure 4a). Intracellular ROS levels and the overlap coefficient, indicating mitochondria/ROS colocalization, were lower in GdP MII oocytes compared with Italian ones (*p* < 0.05 and *p* < 0.001 for ROS levels and overlap coefficient, respectively; Figure 4b,c) whereas no differences were found compared with oocytes of the European sheep population.

### 3.4. Vitrification of GdP Pre-Pubertal Lambs’ Immature COCs Reduce Their Meiotic Maturation after IVM

**Figure 5:**
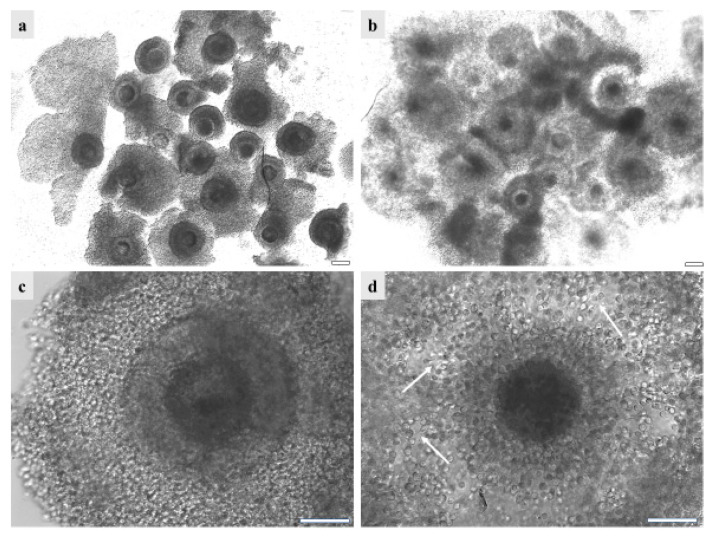
GdP COCs after vitrification/warming as observed (**a**,**c**) before and (**b**,**d**) after IVM, (**a**,**b**) in groups or (**c**,**d**) individually. It can be seen that, before IVM, the majority of GdPs’ vitrified/warmed COCs show complete compact and multilayered cumulus. After IVM, the majority of GdP vitrified/warmed COCs show regular cumulus expansion, with individually visible cumulus cells and their cytoplasmic protrusions. Scale bars represent 40 µm. The arrows indicate the intact cytoplasmic protusions of cumulus cells, after COC vitrification/warming and IVM.

With the aim of developing a germplasm cryobank, the effects of vitrification on GdP immature COCs were evaluated. Indeed, in biodiversity-conservation programs, the development of cryopreservation strategies using immature COCs represents the only method for female germplasm rescue when activities are performed in areas remote from an equipped reproductive biotechnology laboratory. GdP and other pre-pubertal lamb COCs, in six to ten independent runs, were cryopreserved by vitrification. Vitrified COCs were subsequently warmed and subjected to IVM. After culture, cumulus expansion and oocyte maturation rates were compared between the fresh and vitrified samples of each population group (GdP, Italian, and European). In all three sheep populations, the cumulus-expansion rate was significantly reduced upon vitrification (*p* < 0.00001 for all groups, between fresh and vitrified/warmed samples; Table 3). Figure 5 shows GdP COCs after vitrification/warming as observed before (Figure 5a,c) and after (Figure 5b,d) IVM. At warming, the majority of COCs displayed complete compact and multilayered cumulus. After IVM, the majority of GdP vitrified/warmed COCs underwent regular cumulus expansion even though some of them still showed compact or partially/completely removed cumulus. Moreover, cumulus cells maintained the integrity of their cytoplasmic protrusions, which are very important structures for cell-to-cell and cell-to-oocyte communications. In addition, for all sheep population groups, vitrified/warmed (V/W) COCs showed a significantly reduced maturation rate, and corresponding increased rates of oocytes remained at the GV stage (*p* < 0.00001; Table 3).

### 3.5. Vitrification of GdP Pre-Pubertal Lambs’ Immature COCs Does Not Affect Their Bioenergetic/Oxidative Status after IVM

**Figure 6:**
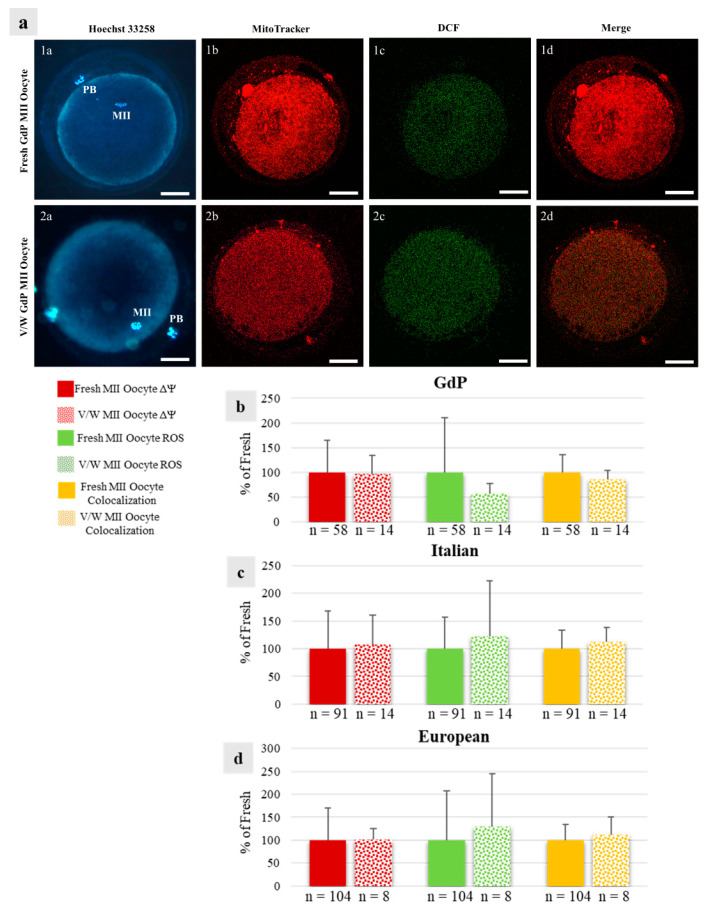
Effects of immature COC vitrification on bioenergetic/oxidative parameters in GdP MII oocytes: (**a**) Photomicrographs showing representative images of one fresh (lane 1) and one vitrified/warmed (V/W) (lane 2) GdP oocyte. Corresponding epifluorescence images showing nuclear chromatin configuration (column **a**: Hoechst 33258) and confocal images showing the mitochondrial distribution pattern and activity (column **b**: MitoTracker Orange), intracellular ROS localization and levels (column **c**: H_2_DCF-DA), and mitochondria/ROS colocalization (column **d**: Merge). Confocal images were taken at the oocyte equatorial plane. Scale bars represent 40 µm. (**b**–**d**) Quantification data of mitochondrial activity (ΔΨ), intracellular reactive oxygen species (ROS) levels, and mitochondria/ROS colocalization in GdP MII oocytes, obtained after IVM of fresh and V/W COCs, compared with Italian and European ones. Within each sheep population, values of V/W oocytes are presented as a percentage of the signal of fresh samples. Means ± SD of fluorescence intensity of MitoTracker Orange CMTM Ros, DCF, and overlap coefficient are presented, respectively. The numbers of analyzed oocytes per sheep population are indicated at the bottom of each bar. Unpaired Student’s *t*-test: not significant.

The rate of GdP MII oocytes showing P/S mitochondrial distribution patterns did not differ between fresh and vitrified oocytes, indicating that oocyte cytoplasmic maturation was maintained after COC vitrification. This was also observed in oocytes of European breeds but not in Italian ones, in which this percentage was significantly lower (*p* < 0.05; Table 3). In COCs of all three sheep populations, bioenergetic/oxidative quantification parameters did not vary based on the vitrification procedure as no differences were observed for mitochondria activity, ROS levels, or overlap coefficient between fresh and vitrified oocytes. Figure 6 shows representative photomicrographs of GdP MII oocytes obtained after IVM of fresh and vitrified/warmed COCs and observed for nuclear chromatin, mitochondria pattern and activity, intracellular ROS localization and levels, and mitochondria/ROS colocalization (Figure 6a) and quantification analysis of the effects of vitrification/warming on oocyte bioenergetic/oxidative status (Figure 6b–d).

## 4. Discussion

Local breeds are important for the area in which they are reared, combining their adaptation and resilience to the territory with the production of unique and inimitable typical products [4]. Moreover, autochthonous breeds represent the historical and cultural heritage of their territory and inhabitants [1]. However, these breeds are facing numeric reduction and are at risk of extinction. Too often, the reason for such a reduction is claimed to be low productivity. However, most of the time, it is the lack of selection programs and the traditional breeding system that impair the obtainment of higher production. The need to retrieve, preserve, and enhance local breeds goes through the application of genetic improvement and assisted reproductive technologies such as ultrasonography, livestock precision farming tools, and biotechnological approaches.

Ultrasonography (US) is a fast (less than one minute for ewes is needed), non-invasive tool that helps in the identification of reproductive failure with large advances compared with the traditional breeding system, where breeders notice the nonpregnant animal only at lambing. Therefore, US could help in the evaluation and optimization of animals’ reproductive performances, thus supporting animal production in marginal areas. Observed PRs are far from the ideal situation, where more than 85% of animals should be pregnant. Due to the problem of retrieving data about sheep reared under a traditional system, it is difficult to explain the reason for such low PRs. Some of the ewes could have been too old for breeding or were inserted into the mating group without a complete recovering after parturition. The use of livestock precision farming tools, such as a real-time database (e.g., SementusaTech^®^) would allow farmers to keep track and easily monitor the reproductive efficiency of each head of the flock using a smartphone [64]. The nutritional and health management of animals and BCS evaluation are key aspects of reproductive efficiency; hence, the lower BCS in nonpregnant ewes was an expected result. However, the overall improvement in the BCS from July to February, not followed by an improvement in the PRs or tPR, might indicate that other factors are affecting the reproductive efficiency of ewes.

The IVM culture of GdP COCs highlighted successful meiosis resumption and showed that nuclear and cytoplasmic maturation were acquired. Indeed, GdP COCs showed good cumulus expansion and maturation rates compared to the oocytes of commercial breeds from Italy or Europe. Moreover, no statistically significant difference was observed in the rate of matured oocytes exhibiting a P/S mitochondrial distribution pattern, indicating that in our culture, most MII oocytes reached cytoplasmic maturation, regardless of the sheep’s breed and origin. Interestingly, oocyte ΔΨ, ROS levels, and he overlap coefficient were shown to be lower in GdPs, usually reared under pasture-based farming systems, compared to commercial sheep breeds, mainly managed under semi-intensive practices with feeding based on industrial fodder and concentrates [4]. Some studies have investigated the correlation between oocyte bioenergetic–oxidative status and unbalanced nutritional intakes in animal models, showing that high-fat, high-fat/high-sugar, or low0protein dietary regimes could increase ROS production and alter oocytes’ mitochondrial membrane potential [65,66,67,68,69]. In conclusion, GdPs’ pre-pubertal lamb COCs showed promising in vitro reproductive potential. Further studies will be needed to evaluate how bioenergetic–oxidative status could influence embryos’ developmental competence for GdP oocytes.

In order to counteract genetic erosion and the possible extinction of this local breed of millenary tradition in the territory of Southern Italy, it is necessary to carry out and implement conservation strategies. According to the DAD-IS database by FAO, cryopreserved semen samples from GdP rams are available; however, there is a total absence of oocytes and embryos. Therefore, this study represents the first attempt to cryopreserve GdP oocytes, using female pre-pubertal lambs as donors, with the aim of developing a germplasm cryobank of this local sheep breed. Immature COC vitrification in endangered species conservation programs represents a versatile and strategic tool, allowing female germplasm to be recovered in large quantities, directly in farms and slaughterhouses located in marginal areas, where these animals are normally bred. Our data are in agreement with most previous studies in adult sheep in which it was found that the vitrification of immature COCs significantly affected cumulus expansion [14,15] and the oocyte maturation rate [12,14,15,17,20,70]. This result was associated with a statistically significant increase in the rate of oocytes arrested at the GV stage [12,14,15,20], regardless of the procedure used (conventional, direct, or on a solid surface) and the device (conventional straw, open pulled straw, cryotop, or cryoloop). Other studies refer to comparable, or even low, rates of matured oocytes between V/W and fresh COCs [22,71,72]. In line with these observations, we found no differences in the maturation rate after using OPS or Rapid-i vitrification devices, both in GdP samples and those of all examined ovine breeds. To date, only one study has reported on pre-pubertal lambs [53] in which the results were similar to those obtained in the present study. To the best of our knowledge, other studies in pre-pubertal subjects are reported only in pigs to date. In this species, V/W immature COCs from pre-pubertal gilts were reported to be significantly affected in their maturation rate after vitrification [33,73], even though later studies reported the fertilization, embryo development [74], and generation of live piglets [33] using V/W immature COCs. Finally, there is only one study on the vitrification of immature COCs applied to local breeds. This study was performed in the Vietnamese Ban Pig, and there was no statistically significant difference in the maturation rate observed after vitrification between the Ban Pig and commercially slaughtered hybrid pigs [54]. Vitrification is known to induce oxidative stress through ROS overproduction, membrane lipid peroxidation, amino acid and nucleic acid oxidation, gene expression alteration, and mitochondrial damage, resulting in cell apoptosis [75]. In our study, although the maturation rate was low after IVM of V/W immature COCs, compared with their fresh counterparts, the cytoplasmic maturation of MII oocytes, expressed as the P/S mitochondrial distribution pattern rate and mitochondria activity, ROS levels, and overlap coefficient quantifications, resulted in preserved COCs comparable to fresh ones. To the best of our knowledge, this is the first study in sheep evaluating cytoplasmic maturation, expressed as bioenergetic/oxidative status, in mature oocytes after the IVM of vitrified immature COCs. Further studies are needed to improve the vitrification procedure and/or IVM conditions with the aim of developing a cryobank of the local sheep breed GdP.

## 5. Conclusions

In conclusion, this study represents the first attempt to establish collaborations with Apulian farms that are located in marginal areas and still use the traditional management of reproductive activity for the autochthonous breed GdP, to push them to modern practices of assisted reproduction and in situ and ex situ conservation strategies. The proposal to monitor the reproductive efficiency on the farm provides the benefits of maintaining high levels of fertility and reproductive health in a GdP flock, and the proposal to develop a cryobank of female germplasm could contribute to maintaining and preserving GdP genetic diversity. Indeed, the present study showed that the ovaries of pre-pubertal lambs slaughtered for food purposes can be used to recover oocytes without interfering with productive and reproductive activities on the farm. After animal genotyping and oocyte vitrification, these cells can be used with fresh or cryopreserved GdP sperm for the in vitro production of embryos. Such strategies, in association with collecting, freezing, and using semen, could allow farmers to plan the maintenance or expansion of the number of animals with a controlled and reducible environmental impact. Flock monitoring and reproductive biotechnologies could also constitute the basis of genetic-improvement strategies in this local breed for its valuable products [76,77]. The following final objectives of the project were reached: (1) the realization of a GdP germplasm cryobank with a sustainable cost that is useful to contain its genetic erosion; (2) the training of reproductive clinicians and biotechnologists in the Apulia region capable of operating in technological, regulatory, and managerial aspects in the conservation of native livestock germplasm; and (3) the enhancement of Apulian livestock farms rearing the local sheep breed GdP.

## Figures and Tables

**Table 1:** Clinical examinations in one Gentile di Puglia (GdP) flock reared in a pilot farm in the Apulia region: pregnancy rates and body condition scores (BCS).

Time of Clinical Examination	N. of Visited Ewes	N. of Pregnant Ewes (%)	N. of Ewes with Twin Pregnancy (%)	BCS of Pregnant Ewes Mean ± s.d.	BCS of Nonpregnant Ewes Mean ± s.d.
July 2021	232	136 (59)	25 (18) ^a^	2.69 ± 0.28 ^aA^	2.42 ± 0.48 ^aD^
November 2021	78	51 (65) ^a^	6 (12)	2.76 ± 0.22 ^A^	2.6 ± 0.31 ^B^
February 2022	303	157 (52) ^b^	15 (10) ^b^	2.82 ± 0.28 ^cA^	2.65 ± 0.32 ^eE^

Table legend: BCS = Body condition score; Chi-Square test: Within the “Pregnant Ewes” and “Ewes with Twin Pregnancy” columns, comparisons were made between the July 2021, November 2021, and February 2022 clinical evaluation times; a, b = *p* < 0.05; one-way ANOVA, followed by Tukey’s Multiple Comparison Test. Within the “BCS of Pregnant Ewes” and “BCS of nonpregnant ewes” columns, comparisons were made among the three time periods: a, c = *p* < 0.01; a, e = *p* < 0.0001. Unpaired Student’s *t*-test: between the “BCS of Pregnant Ewes” and “BCS of nonpregnant ewes” columns, comparisons were made for each time of clinical examination: A, B = *p* < 0.05; A, D = *p* < 0.001; A, E = *p* < 0.0001.

**Table 2:** Nuclear and cytoplasmic parameters of Gentile di Puglia (GdP) pre-pubertal lamb oocytes after in vitro maturation (IVM) compared with commercial sheep breeds.

Sheep Population	N. of Cultured COCs (Replicates)	Cumulus Expansion Rate N. (%)	N. of Evaluated COCs	Nuclear Chromatin Configurations N. (%)				P/S Mitochondrial Distribution Pattern N. (%)
				GV	MI to TI	MII+PB	Abnormal	
GdP	176 (7)	172 (97.7) ^a^	172	16 (9.3) ^a^	20 (11.6)	115 (66.9) ^a^	21 (12.2)	53/113 (46.9)
Italian	188 (8)	163 (86.7) ^d^	178	50 (28.1) ^e^	16 (9.0)	88 (49.4) ^d^	24 (13.5)	38/64 (59.4)
European	201 (8)	180 (89.6) ^b^	188	41 (21.8) ^d^	14 (7.4)	105 (55.9) ^b^	28 (14.9)	57/105 (54.3)

Table legend: COC = Cumulus–oocyte complex; GV = Germinal vesicle; M = Metaphase; PB = Polar body; P/S = Perinuclear/Subcortical. Chi-Square test: within each column, comparisons were made between GdP and commercial, Italian, or European, groups: a, b = *p* < 0.05; a, d = *p* < 0.001; a, e = *p*< 0.00001.

**Table 3:** Nuclear and cytoplasmic parameters of Gentile di Puglia (GdP) pre-pubertal lamb vitrified/warmed immature cumulus–oocyte complexes (COCs) after IVM compared with commercial sheep breeds.

Sheep Population	COC Vitrification	N. of Cultured COCs (Replicates)	Cumulus Expansion Rate N. (%)	N. of Evaluated Oocytes	Nuclear Chromatin Configurations N. (%)				P/S Mitochondrial Distribution Pattern
					GV	MI to TI	MII+PB	Abnormal	
GdP	−	146 (6)	142 (97.3) ^a^	135	25 (18.5) ^a^	23 (17.0)	71 (52.6) ^a^	16 (11.9)	38/58 (65.5)
	+	131 (6)	79 (60.3) ^e^	120	72 (60.0) ^e^	14 (11.7)	14 (11.7) ^e^	20 (16.6)	7/14 (50.0)
Italian	−	163 (7)	160 (98.2) ^a^	158	10 (6.3) ^a^	26 (16.5)	92 (58.2) ^a^	30 (19.0)	38/91 (41.8) ^a^
	+	127 (7)	64 (50.4) ^e^	116	60 (51.7) ^e^	21 (18.1)	14 (12.1) ^e^	21 (18.1)	1/14 (7.1) ^b^
European	−	200 (10)	191 (95.5) ^a^	192	25 (13.0) ^a^	32 (16.7)	105 (54.7) ^a^	30 (15.6)	60/104 (57.7)
	+	146 (10)	51 (34.9) ^e^	140	82 (58.6) ^e^	16 (11.4)	11 (7.9) ^e^	31 (22.1)	4/8 (50.0)

Table legend: COC = Cumulus–oocyte complex; GV = Germinal vesicle; M = Metaphase; PB = Polar body; P/S = Perinuclear/Subcortical. Chi-Square test: within each group, comparisons were made between fresh and vitrified/warmed COCs, after IVM: a, b = *p* < 0.05; a, e = *p* < 0.00001.

## Data Availability

The data presented in this study are available on request from the corresponding author.
